# Supplementary material for: How Effective Is Environmental Protection for Ensuring the Vitality of Wild Orchid Species? A Case Study of a Protected Area in Italy
Source: Plants (Basel). 2024 Feb 23;13(5):610. doi: 10.3390/plants13050610 (PMC10935256; doi:10.3390/plants13050610)
Supplement: Supplementary file 1 [file plants-13-00610-s001.zip › plants-2825204-supplementary.pdf]

Supplementary Table S1 – Brief summary of top-soil chemistry at the sampling sites.

Species	Protection	pH	Conductivity	Salinity	N-NH_4	N-NO_3	P-PO_4
O. sphegodes	P	6.23	348.40	0.1	2.99	16.46	4.55
	NP	6.19	267.10	0.05	0.64	46.73	11.85
A. morio	P	6.42	458.70	0.16	3.52	19.86	4.81
	NP	6.22	201.20	0.02	0.96	28.77	5.27
A. pyramidalis	P	5.91	164.57	0.00	2.11	10.79	4.12
	NP	6.23	197.40	0.00	0.38	12.47	3.68
A. coriophora	P	6.20	370.57	0.11	3.27	17.06	4.81
	NP	6.26	160.84	0.00	2.91	7.12	3.65

Supplementary Table S2 - ANOVAs of the risk categories (1: absent; 2: weak; 3: moderate; 4: high). The asterisks indicate significant ($p < 0.05$) differences between protected (P) and non-protected (NP) sites.

Species	Protection	Mowing	Waste	Herbivory	IAS
O. sphegodes	P	3.00±0.58	2.00±0.58	1.67±0.67	2.33±0.67
	NP	3.00±0.58	1.33±0.33	2.00±0.58	3.33±0.58
A. morio	P	1.67±0.67	1.67±0.67	1.67±0.67	2.33±0.67
	NP	2.33±0.89	2.67±0.89	2.67±0.67	3.33±0.67
A. pyramidalis	P	2.00±1.00	1.67±0.67	1.33±0.33	1.67±0.33
	NP	1.67±0.67	2.33±0.67	1.33±0.33	2.33±0.66
A. coriophora	P	2.67±0.89	2.67±0.89	1.00±0.00	2.00±0.58
	NP	2.75±0.75	2.75±0.48	2.00±0.70*	4.00±0.00*

Supplementary Figure S1 – Pictures of orchid populations at protected (left) and non-protected (right) sites.

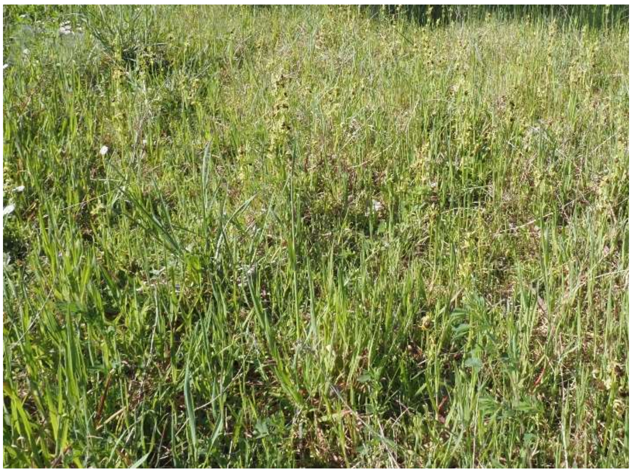

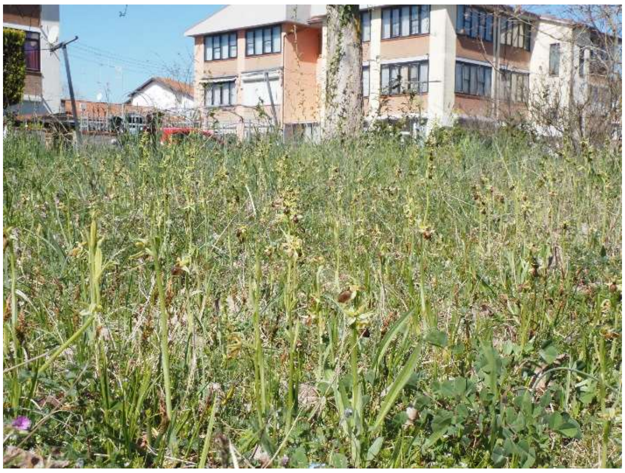

Ophrys sphegodes

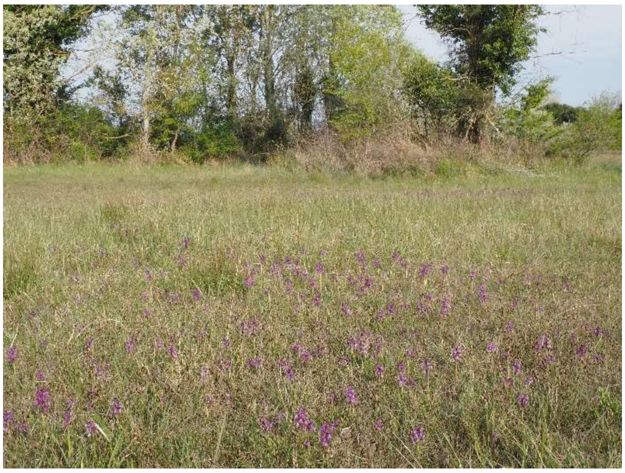

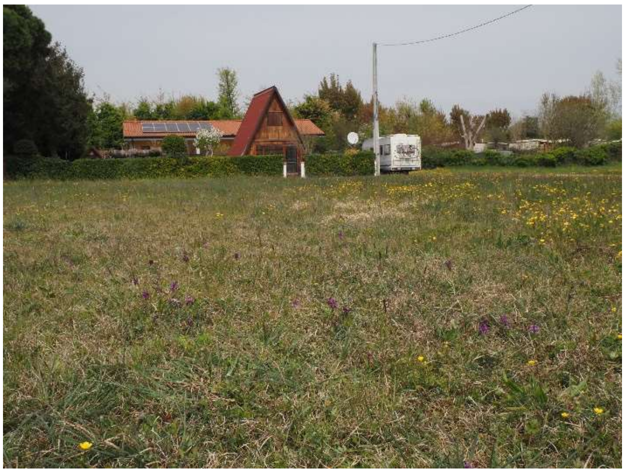

Anacamptis morio

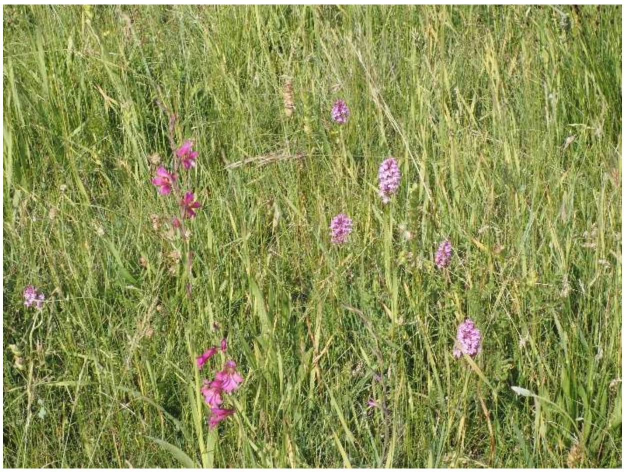

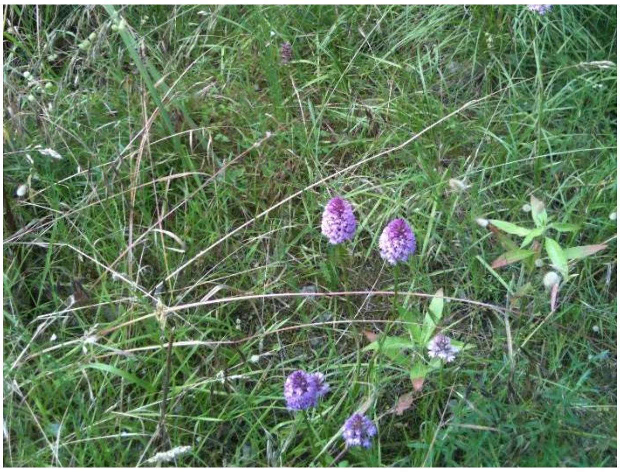

Anacamptis pyramidalis

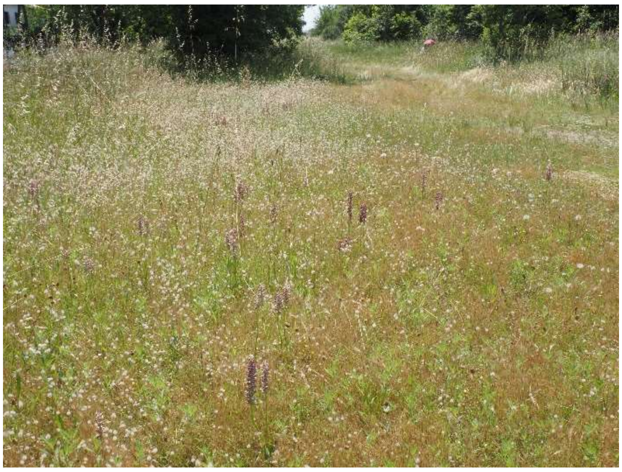

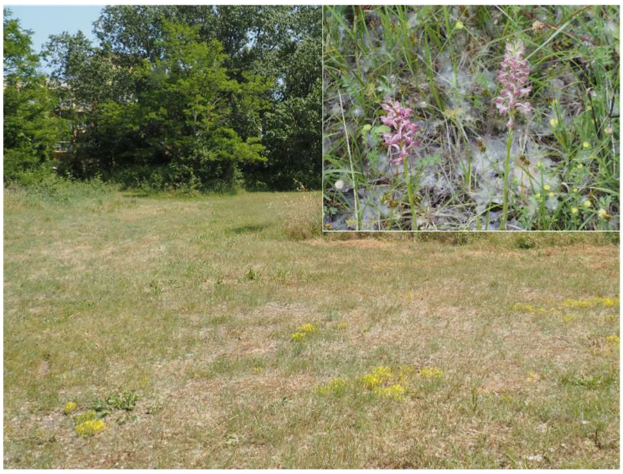

Anacamptis coriophora
